# Metastatic Bone Epithelioid Hemangioendothelioma: A Rare Case Report

**DOI:** 10.7759/cureus.117792

**Published:** 2026-10-12

**Authors:** Hasnae Haouzi, Othmane Zouiten, Leila Afani, Mohamed El Fadli, Rhizlane Belbaraka

**Affiliations:** 1 Department of Medical Oncology, Hôpital Universitaire de Marrakech, Marrakech, MAR; 2 Department of Medical Oncology, Mohammed VI University Hospital of Marrakech, Marrakech, MAR; 3 Department of Medical Oncology, Centre Hospitalier Universitaire (CHU) Mohammed VI, Marrakech, MAR; 4 Department of Medical Oncology, Mohammed VI University Hospital, Marrakech, MAR; 5 Department of Medical Oncology, Hôpital Universitaire de Marrakech, Faculté de Médecine et de Pharmacie, Université Cadi Ayyad, Marrakech, MAR

**Keywords:** anatomopathology diagnosis, bone, case report, chemotherapy agents, epithelioid hemangioendothelioma, metastasis, molecular testing, tumor

## Abstract

Epithelioid hemangioendothelioma (EHE) is an exceptionally rare vascular neoplasm that often represents a diagnostic challenge. It predominantly affects women and most commonly involves the liver, lungs, and bones. To date, no specific predisposing factors have been clearly identified. Data regarding the management of metastatic EHE remain limited, and standardized therapeutic strategies are lacking. At early stages, surgical resection, including amputation when necessary, with or without radiotherapy, remains the mainstay of treatment to prevent disease dissemination. The medical oncologist plays a crucial role in this rare entity, especially in advanced stages, by guiding a multidisciplinary, individualized approach. We report the case of a 61-year-old patient who presented with painful swelling on the lateral aspect of the right knee. Histopathological examination of bone and soft tissue biopsies favored the diagnosis of a high-risk vascular tumor consistent with epithelioid hemangioendothelioma. Staging investigations revealed widespread metastatic disease involving the liver, bones, lungs, and intestinal tract. This case highlights the aggressive potential of EHE and underscores the need for early oncological involvement and the development of evidence-based therapeutic strategies. Despite its rarity, advancing clinical research and establishing treatment recommendations are essential to improve outcomes in patients with advanced EHE.

## Introduction

Epithelioid hemangioendothelioma (EHE) is a rare vascular tumor of intermediate malignancy, frequently presenting as multifocal disease [1]. Classified within the sarcoma spectrum, it is characterized by epithelioid endothelial cells with intracytoplasmic vacuoles and variable vasoformative differentiation [2]. Its biological behavior is highly heterogeneous, ranging from indolent disease to locally aggressive growth and metastatic progression [3].

EHE can arise in several anatomical sites, including the liver, lungs, soft tissues, and bone. Osseous involvement is uncommon and may present with pain, swelling, or pathological fracture, often creating diagnostic and therapeutic challenges [1,4]. Histological diagnosis may be difficult because of its morphological overlap with other epithelioid neoplasms, particularly high-grade angiosarcoma and metastatic carcinoma [2].

A key molecular hallmark of EHE is the WWTR1-CAMTA1 gene fusion, which is highly characteristic of conventional EHE and helps distinguish it from other vascular neoplasms [3]. Due to its rarity and unpredictable clinical course, treatment should be individualized according to disease extent, anatomical site, symptoms, and tumor progression. Surgical management remains an important option for localized bone disease, while advanced or unresectable disease may require systemic or other local approaches [3,4].

In this report, we describe the clinical, radiological, and histopathological features of a metastatic tibial EHE, with a particular focus on its oncological management and the challenges posed by advanced-stage disease.

## Case presentation

We report the case of a 61-year-old Moroccan man with no known medical history, presenting with swelling of the right knee and pain, causing total functional impairment of the leg beginning six months prior. On clinical examination, the patient was in generally good health, with an Eastern Cooperative Oncology Group (ECOG) performance status of 2 due to right-limb pain. Clinical examination of the affected leg revealed a large knee with collateral venous circulation. Palpation revealed a hard and painful region of about 15 cm (Figure 1). There were no palpable lymph nodes in the groin, femoral, or popliteal areas. The definitive diagnosis was difficult to establish.

**Figure 1:**
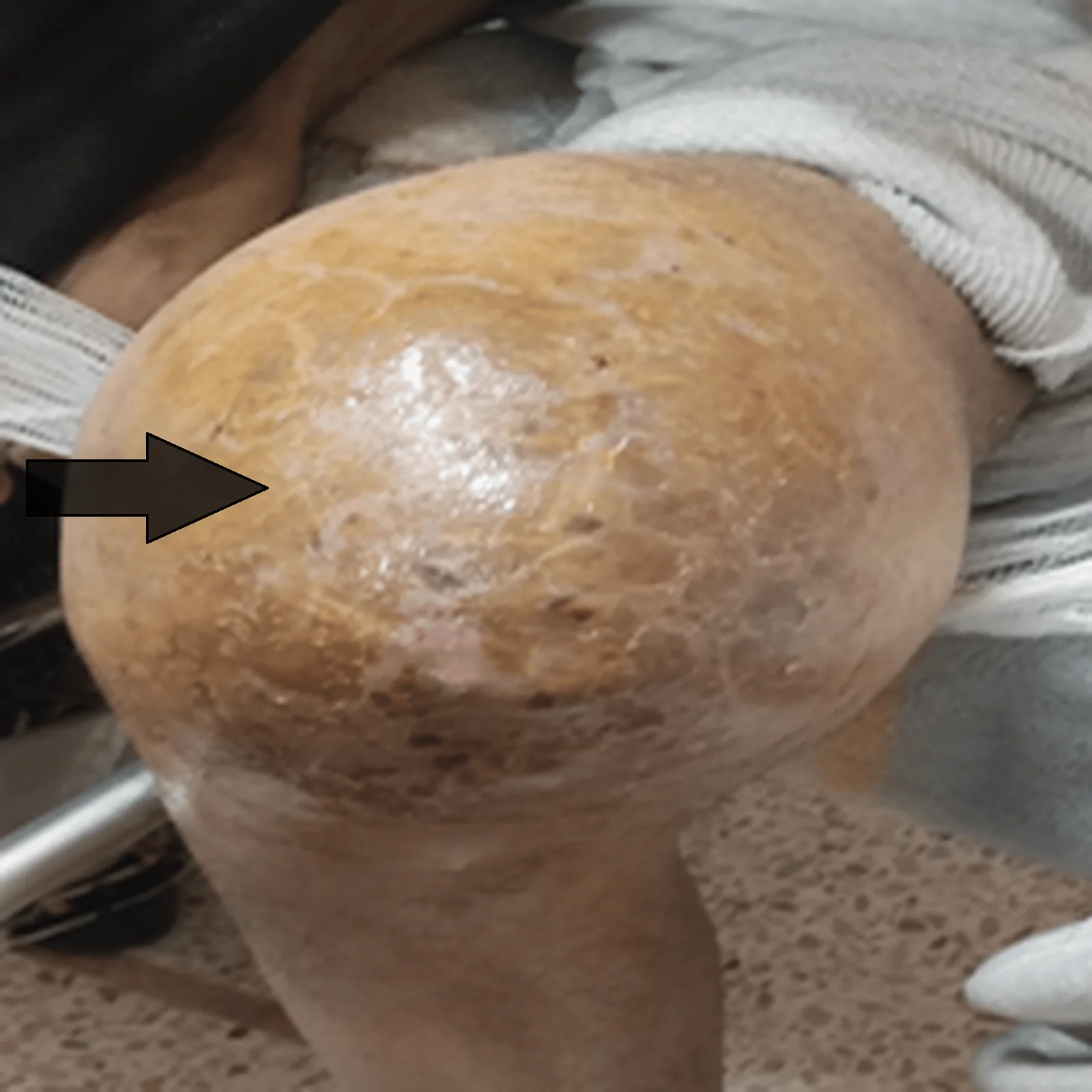
A 61-year-old patient presenting with a swelling of the right knee measuring 15 cm.

Several paraclinical tests were carried out to a final clinical diagnosis. A magnetic resonance imaging (MRI) of the right knee was performed and showed a well-defined soft tissue, with no cortical rupture or medullary change. A cervical, thoracic, and abdominopelvic CT scan revealed lung, liver, and bone metastases.

Bone scintigraphy further confirmed skeletal involvement, showing abnormal uptake in the distal extremity of the right femur and in the manubrium of the sternum. These lesions were characterized by areas of photopenia, indicating absence of tracer fixation, consistent with aggressive bone involvement (Figures 2a-2c).

**Figure 2:**
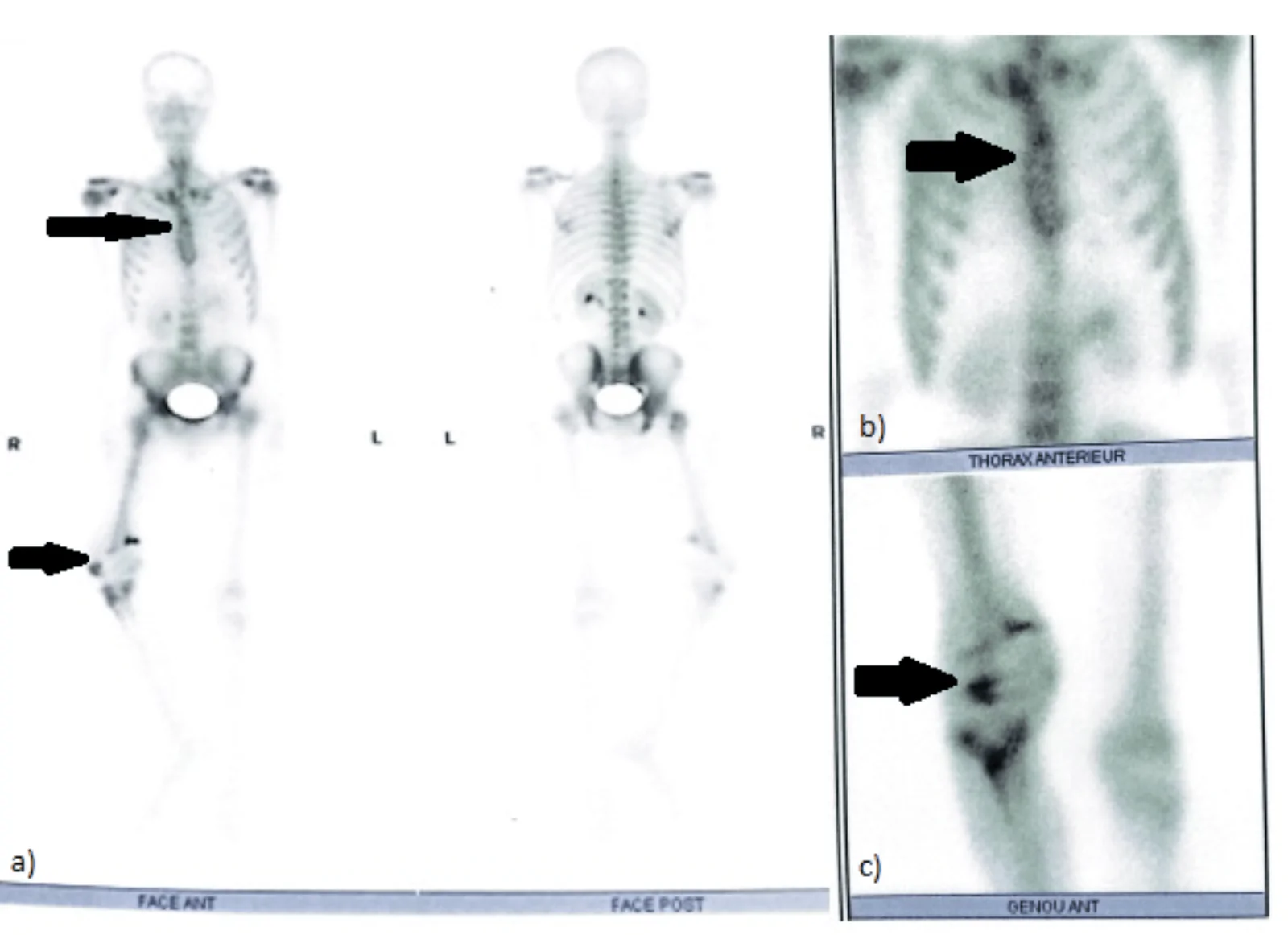
A photopenic area (absence of radiotracer uptake) was observed at the level of the left lateral aspect of the sternal manubrium. In addition, there was deformity of the distal right femur with heterogeneous tracer uptake, associated with increased uptake at the proximal tibia. Figures (a)-(c) show bone scintigraphy findings demonstrating abnormal uptake in the distal right femur and the manubrium of the sternum, with areas of photopenia consistent with aggressive bone involvement.

Histopathological examination was performed on fragmented specimens obtained from the osseous and synovial/adjacent soft-tissue components of the lesion (Figure 3). Hematoxylin and eosin-stained sections revealed a malignant sarcomatous neoplasm composed of large atypical cells arranged in densely cellular solid sheets, with no evidence of acinar, glandular, or epithelial differentiation. The tumor cells displayed elongated nuclei with prominent nucleoli, focal marked nuclear pleomorphism, and brisk mitotic activity. The background stroma was hyalinized and non-inflammatory, and the vascular structures showed thickened walls. Areas of tumor necrosis were also present. Based on this high-grade sarcomatous morphology, an immunohistochemical study was performed to further characterize the tumor phenotype and refine the differential diagnosis.

**Figure 3:**
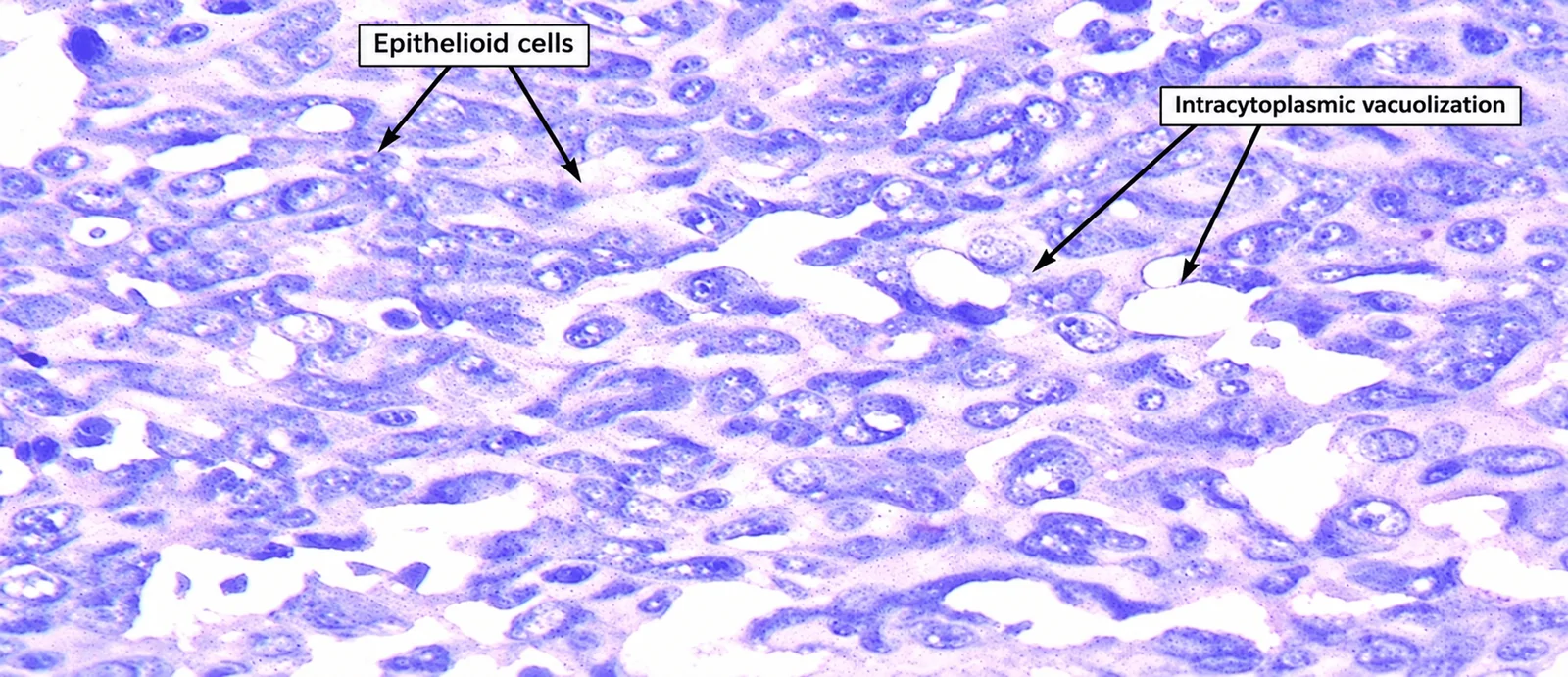
Histopathological image showing a proliferation of epithelioid cells with prominent intracytoplasmic vacuolization, characteristic of epithelioid hemangioendothelioma.

The tumor cells showed strong positivity for CD31 (Figure 4), CD34, and vimentin, with focal staining for CD99. The Ki-67 proliferation index was estimated at approximately 10% of tumor cells. In contrast, the tumor cells were negative for pancytokeratin, epithelial membrane antigen (EMA), Bcl-2, calretinin, and S-100 protein. The immunohistochemical profile supports the diagnosis of a malignant vascular tumor, most consistent with epithelioid hemangioendothelioma.

**Figure 4:**
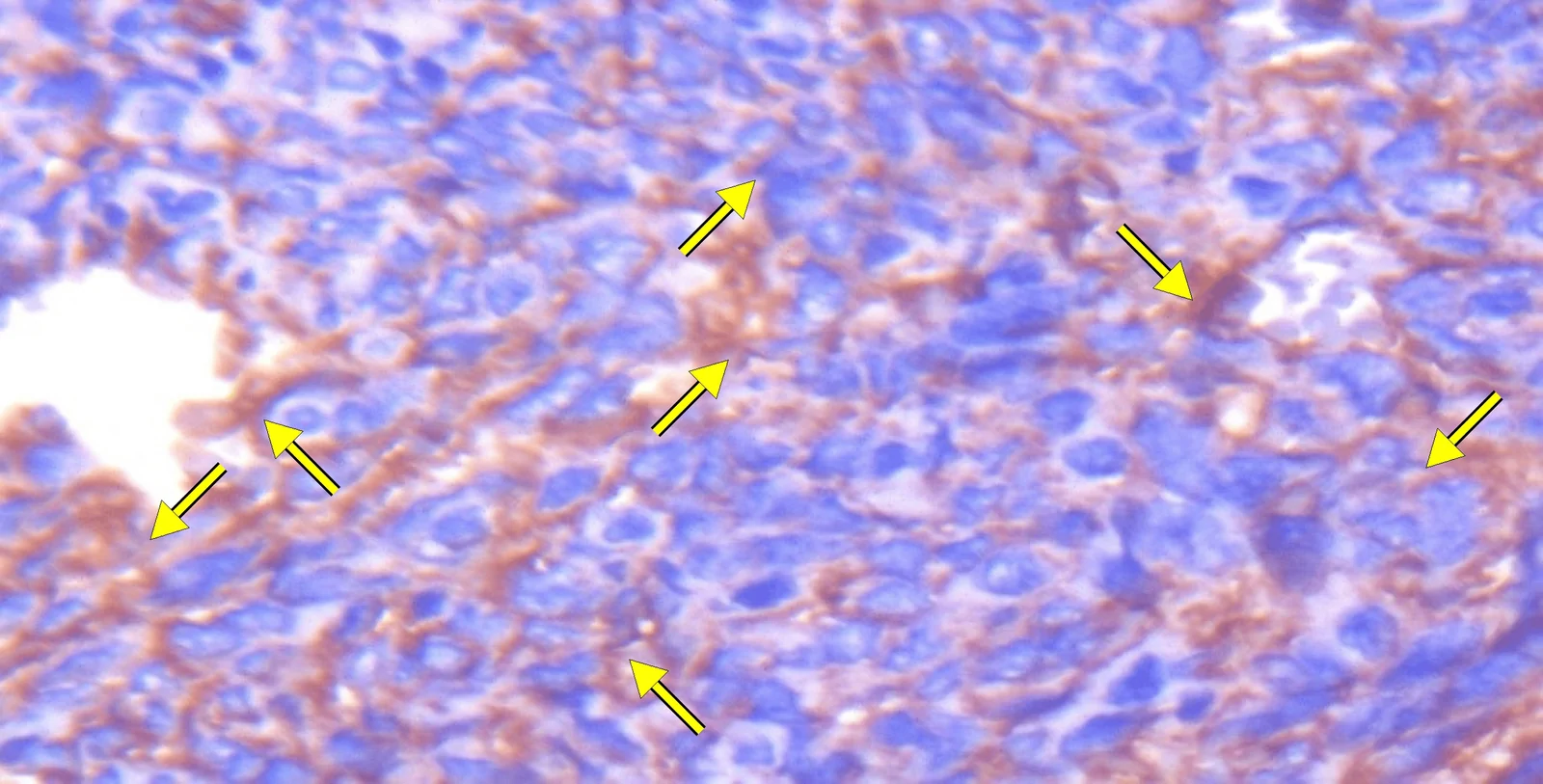
The tumor cells show strong and diffuse membranous immunoreactivity, consistent with endothelial differentiation, in keeping with CD31 positivity.

Following evaluation, the patient was found to have a satisfactory functional and biological status, allowing consideration of systemic therapy. After discussion at a multidisciplinary tumor board, first-line treatment with weekly paclitaxel (80 mg/m²) was recommended.

However, before initiation of chemotherapy, the patient developed significant anemia associated with active bleeding from the knee lesion, prompting urgent medical consultation. Due to this deterioration and the severity of anemia, systemic treatment could not be initiated, and the patient did not receive any chemotherapy cycles.

Radiotherapy was discussed for the patient; however, the multidisciplinary tumor board decided against it due to the increased risk of vascular complications.

Despite supportive care, the patient's general condition progressively worsened. He subsequently died as a result of overall clinical deterioration related to disease progression.

## Discussion

Epithelioid hemangioendothelioma (EHE) is an exceptionally rare malignant vascular neoplasm, with an estimated incidence of less than 1 case per 100,000 individuals [1]. First described by Weiss and Enzinger in 1982, EHE is characterized by an intermediate biological behavior between epithelioid hemangioma and angiosarcoma [2]. It predominantly affects young to middle-aged adults, although it can occur across a wide age range, with a slight female predominance reported in some series [3]. The liver, lungs, soft tissues, and bone are among the most commonly involved sites, and multifocal disease involving several organs may occur [3]. Bone involvement is uncommon and may present as either solitary or multifocal disease [4]. No specific predisposing factors have been clearly established. Molecularly, EHE is characterized in approximately 90% of cases by the WWTR1-CAMTA1 gene fusion, which represents a key diagnostic hallmark [5,6]. A smaller subset, often occurring in younger patients, harbors a YAP1-TFE3 fusion [6,7].

Clinically, osseous EHE commonly presents with nonspecific symptoms, particularly persistent localized pain, swelling, and functional impairment, which may contribute to delayed diagnosis [4]. In our case, a 61-year-old man presented with a painful swelling of the right knee that had progressed over six months, resulting in complete functional impairment. Clinical examination revealed a large mass measuring approximately 15 cm, associated with visible collateral venous circulation. EHE may follow an indolent course in some patients, whereas others develop multifocal or metastatic disease. The lungs, liver, and bones are frequently involved in patients with systemic disease [3,4], as illustrated by our patient, who presented with hepatic, pulmonary, osseous, and intestinal metastases.

Imaging findings of osseous EHE are nonspecific. Radiographs and CT typically show osteolytic lesions that may involve cortical destruction and extend into surrounding soft tissues [4]. MRI is particularly useful for evaluating the extent of bone and soft-tissue involvement and may demonstrate heterogeneous signal intensity and enhancement related to the vascular nature of the tumor [4]. Whole-body MRI or fluorodeoxyglucose positron emission tomography/computed tomography (FDG-PET/CT) may be useful for identifying multifocal and metastatic disease, while bone scintigraphy can be considered when these modalities are unavailable [3]. In our case, MRI demonstrated a well-defined soft-tissue lesion without evidence of cortical rupture or medullary involvement.

Histopathological examination remains the cornerstone of diagnosis. EHE is characterized by epithelioid cells arranged in cords, strands, or small nests within a distinctive myxohyaline stroma, with frequent intracytoplasmic vacuoles representing primitive vascular lumina [6]. Immunohistochemistry typically demonstrates endothelial differentiation, with expression of CD31, ERG, CD34, and FLI-1 [6]. Molecular confirmation of WWTR1-CAMTA1 or YAP1-TFE3 fusion can further support the diagnosis and help distinguish EHE from other vascular neoplasms, particularly epithelioid angiosarcoma [6,7]. In our case, the histopathological and immunohistochemical findings were consistent with EHE, with a Ki-67 proliferation index of approximately 10%.

There is currently no standardized treatment strategy for EHE because of its extreme rarity and heterogeneous clinical behavior. For localized and resectable disease, complete surgical resection remains the preferred approach when feasible [3,4]. In selected cases, radiotherapy may be considered for local control when surgery is not feasible or when local recurrence is a concern. In metastatic disease, management should be individualized according to disease extent, symptoms, rate of progression, and organ involvement. For asymptomatic metastatic disease without clear progression, active surveillance may be appropriate, whereas systemic therapy can be considered in patients with progressive, symptomatic, or organ-threatening disease [3]. Available systemic treatments include chemotherapy, anti-angiogenic agents, and other targeted approaches, although evidence remains limited and is mainly derived from retrospective series and case reports [3]. In our case, weekly paclitaxel was considered based on the available literature; however, treatment could not be initiated because of severe anemia related to active bleeding from the knee lesion, and the patient subsequently died from clinical deterioration related to disease progression.

Given the vascular nature of EHE and the potential involvement of major vessels, the use of radiotherapy requires careful consideration. Radiation-induced vascular injury may result in progressive structural changes of the vessel wall and, in rare cases, pseudoaneurysm formation and vascular rupture [8]. Therefore, in the presence of extensive vascular involvement, the potential risk of severe vascular complications should be carefully weighed when considering radiotherapy. In our case, this potential risk was discussed during the multidisciplinary tumor board and contributed to the decision not to proceed with radiotherapy.

The clinical course of EHE is highly variable, ranging from prolonged indolent disease to aggressive progression with extensive systemic involvement [3,6]. Prognosis appears to depend on disease extent, multifocality, and the presence of visceral involvement, although reliable prognostic factors remain incompletely defined [3,4]. The rarity of metastatic bone EHE and the absence of prospective clinical trials make evidence-based treatment particularly challenging. Management should therefore be individualized and discussed within a multidisciplinary sarcoma team, with referral to specialized centers whenever possible [3]. Further prospective studies and collaborative registries are needed to better define prognostic factors and establish effective systemic treatment strategies for this ultra-rare malignancy.

## Conclusions

Bone epithelioid hemangioendothelioma (EHE) is an exceptionally rare vascular sarcoma. Definitive diagnosis relies on histopathological examination, supported by immunohistochemical and molecular analyses. Due to the very limited number of reported cases of metastatic bone EHE and the absence of clinical studies establishing an optimal treatment strategy, the decision to use paclitaxel in our patient was guided by existing case series.

Although metastatic bone EHE is rare, there is a clear need for standardized systemic therapy protocols to improve patient outcomes and guide clinical management.
